# Retraction Note: SOG1 transcription factor promotes the onset of endoreduplication under salinity stress in Arabidopsis

**DOI:** 10.1038/s41598-024-54831-1

**Published:** 2024-02-21

**Authors:** Kalyan Mahapatra, Sujit Roy

**Affiliations:** Department of Botany, UGC Center for Advanced Studies, The University of Burdwan, Golapbag Campus, Burdwan, West Bengal 713 104 India

Retraction of: *Scientific Reports* 10.1038/s41598-021-91293-1, published online 02 June 2021

The Editors have retracted this Article. After publication, concerns were raised regarding the data presented in the figures. Specifically:

- In Figure 1A, the sog1-6, 0 NaCl (mM), image appears highly similar to the sog1-1, 0 NaCl (mM), panel;
- In Figure 5B, the 150 mM NaCl Wild Type plot image appears highly similar to the Figure 5C Control sog1-1 plot image;
- In Figure 6B, the 50 mM NaCl Wild Type image appears highly similar to the Figure 6D 150 mM NaCl sog1-1 image;
- In Figure 7E, the +NaCl OE-1 image appears highly similar to the +NaCl Wild Type image;
- In Figure 7E, the Control Wild Type image appears highly similar to the Control sog1-6 image;
- In Figure 7E, the +NaCl sog1-6 image appears highly similar to the +NaCl sog1-1 image;
- In Figure 7F, the +NaCl sog1-6 image has elements that appear highly similar to the Control sog1-1 image;
- In Figure 7F, the +NaCl sog1-6 image has elements that appear highly similar to the +NaCl sog1-1 image;
- In Figure 8A, the Control OE-1 image has elements that appear highly similar to the +NaCl OE-1 image;
- In Figure 9E, the OE-1 blot image appears highly similar to background elements in the Figure11B OE-1 blot image.

The Editors therefore no longer have confidence in the data presented.

All authors agree to this retraction.

